# Simultaneous fabrication of line defects-embedded periodic lattice by topographically assisted holographic lithography

**DOI:** 10.1186/1556-276X-6-449

**Published:** 2011-07-12

**Authors:** Byung-Yeon Choi, Yusin Pak, Ki Seok Kim, Kwang-Ho Lee, Gun-Young Jung

**Affiliations:** 1 Department of Materials Science and Engineering Gwangju Institute of Science and Technology (GIST), 261 Cheomdan-gwagiro, Buk-gu, Gwangju 500-712, Republic of Korea

**Keywords:** hot-embossing, holographic lithography, phase-shift lithography, photonic crystal, defect generation

## Abstract

We have demonstrated simultaneous fabrication of designed defects within a periodic structure. For rapid fabrication of periodic structures incorporating nanoscale line-defects at large area, topographically assisted holographic lithography (TAHL) technique, combining the strength of hologram lithography and phase-shift interference, was proposed. Hot-embossing method generated the photoresist patterns with vertical side walls which enabled phase-shift mask effect at the edge of patterns. Embossing temperature and relief height were crucial parameters for the successful TAHL process. Periodic holes with a diameter of 600 nm at a 1 μm-pitch incorporating 250 nm wide line-defects were obtained simultaneously.

## Background

Photonic crystals (PhC) affect light motion due to being periodically comprised of various dielectric materials [1,2]. Photonic band-gap generation within the PhC structures impacts light propagation at certain wavelengths [3] in a similar manner to electronic band-gap within atomic lattice, which governs electron transport in current electronic appliances. Conventional devices using electrons as carriers have approached to their performance limits with scaling down the dimension and/or the continued chip integration; therefore, many scientists believe that light will bring a major breakthrough, being able to travel within a confined dielectric material much faster than the electrons: more information delivery per unit of time [4]. Moreover, energy loss, relative to electrons, can be significantly reduced due to the less inter-photon interferences.

Accordingly, PhC structures with functional defects such as photonic cavities and waveguides have attracted much interest in photonic device fabrication. In particular, researchers have utilized various technologies such as colloidal self-assembly [5], layer-by-layer assembly [6], glancing angle deposition [7] and multi-beam holographic lithography [8] for 3-dimensional (3D) photonic crystal structure fabrication. However, such methods are not intrinsically appropriate for the incorporation of functional defects into the 3D structures. Employment of a sophisticatedly made poly(dimethylsiloxanes) (PDMS) stamp with feature sizes comparable to light wavelength as a diffraction optical element facilitates simultaneous point defect incorporation into the 3D nanostructures [9]. Juntao Li et al. created simultaneous functional defects within the PhC via their multi-beam phases holographic lithography [10] but, the period less than 5 μm and the beam focusing area larger than 100 μm were not realized with this method.

Researchers have developed a series of techniques to incorporate functional defects into 2D and 3D PhCs by means of an elaborative extrinsic lithographic way [11,12]. Loncar et al. [13] and Notomi et al. [14] have demonstrated 2D PhC fabrication as well as nanoscale waveguiding lines simultaneously, called intrinsic defects fabrication, via electron-beam lithography despite of being time-consuming and difficult for large area application. Therefore, the concept of holographic lithography was brought as a breakthrough to generate the periodic structure over large area, replacing the time consuming serial writing process; but, there is still a matter to be solved concerning the creation of functional defects within it.

Cho et al. demonstrated photonic crystal slab waveguides by the combination of holography and photolithography; defect lines were introduced extrinsically into a pre-patterned PhC structure [15]. Although this technique significantly reduced time and expense, problems persisted with functional defects fabrication with a scale of below 2 μm. Sun et al. [16] and Scrimgeour et al. [17] have explored laser direct writing and two-photon laser writing for fabricating functional defects in holographically pre-patterned 2D and 3D PhC lattices, respectively. Although such technologies can lead to nanoscale resolution, they also suffer from time-consuming pixel-by-pixel scanning for the defects incorporation.

Rodgers et al. have demonstrated phase-shift mask effect - that is utilized to generate line-defects in current study - in which a PDMS stamp made a conformal contact to a underlying polymer layer and was subjected to UV exposure [18]. The lights through the PDMS relief was phase-shifted against the lights passing through the air at the PDMS/air boundaries, attenuating the dose to shadow the photoresist (PR) located at the edge of the PDMS reliefs. For our preliminary experiment, this method was innovatively combined with interference lithography (hologram lithography). The transparent PDMS stamp with relief features in contact with the negative tone PR was exposed to interfered laser lights twice with a 90 degree rotation hopefully to generate both the periodic hole structures and line-defects at the PDMS relief edges simultaneously. However, subtle perturbation of light intensity possibly due to the light scattering at the PDMS sagging surface caused failure of obtaining the nanoscale line-defects after development process.

In this communication, we explored a new lithographic technique, topographically assisted holographic lithography (TAHL), in order to generate defect-embedded periodic structures. Prior to holographic lithography, we created the PR patterns by hot-embossing method, replacing the PDMS phase-shift mask, not only to shift the light phase at the edge of relief but also to eliminate previously mentioned unwanted light scattering at the PDMS surface.

## Results and discussion

**Figure 1:**
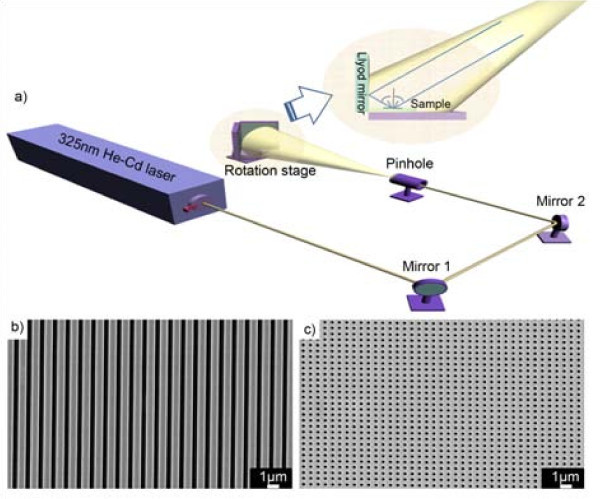
**Hologram lithography**. **(a) **Experimental set-up of hologram lithography. **(b) **FE-SEM images of PR lines fabricated by single exposure and **(c) **periodic PR holes fabricated by double exposure.

Hologram lithography was adopted in this study to generate periodic structure in addition to its outstanding merits, e.g., easy and fast processing, large-area patterning and easy density modulation. Figure 1a illustrates our setup of the holographic lithography with a 325 nm He-Cd laser. The laser beam passed through two consecutive mirrors (1, 2), a spatial filter and finally a pinhole, by which waves were diffracted; the diffracted waves were then propagated toward the sample and a Lloyd mirror. The beam reflected at the Lloyd mirror interfered with the beam directly projected onto the sample at certain incident angle (*θ*), generating sinusoidal intensity distribution on the PR according to the following equation; P = λ/2sin*θ*, where P equals the pitch or lattice constant of a periodic pattern, and λ is the laser wavelength. Figure 1b illustrates 650 nm wide periodic PR lines at a 1 μm pitch fabricated by single exposure with an incident angle of 9.35°. Figure 1c demonstrates periodic PR holes with a diameter of 250 nm at a 600 nm pitch fabricated by double exposure; after first exposure, the sample was rotated by 90°, the second exposure was then performed with an incident angle of 15.72°. Patterns were uniformly generated in up to 2-in. wafer.

**Figure 2:**
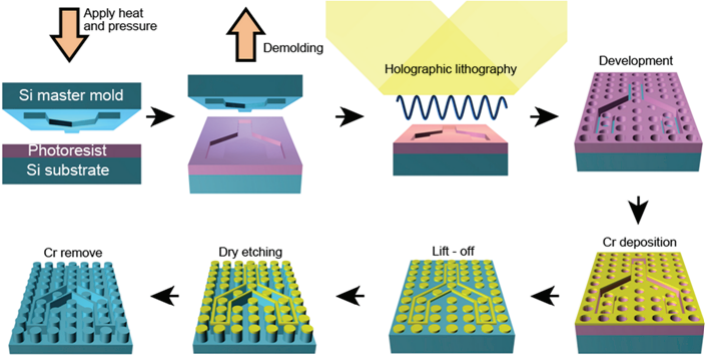
**Schematic illustration of TAHL process for defining line-defects within a periodic structure**.

Figure 2 represents the entire TAHL process. At first, a silicon stamp with features should be fabricated via photolithography. Relief structures with very vertical side walls are prerequisite for the successful phase-shift mask effect. To replicate the stamp features inversely into a PR layer, hot-embossing lithography - in which both pressure and heat were employed as main parameters - was executed. Embossing with external pressure - compared to chemical means such as solvent-assisted embossing [19] - was advantageous in obtaining the vertical side walls, which are very crucial for appropriate phase-shift effect that defines line-defects in the following laser exposure.

**Figure 3:**
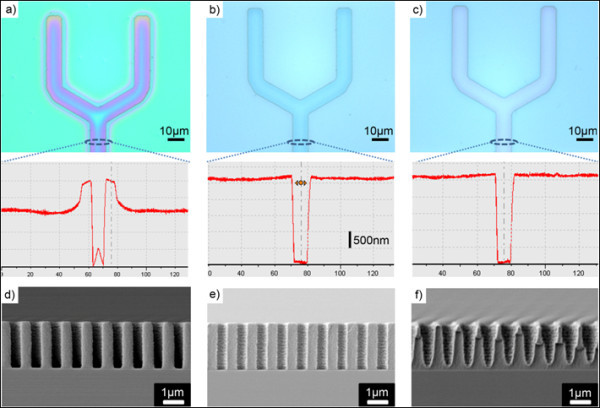
**The effect of temperature on TAHL process**. Microscopic images and surface profiles of the embossed PR patterns at temperatures of **(a) **70°C, **(b) **90°C, and **c) **110°C. FE-SEM cross-sectional views after single interference lithography and subsequent development with the embossed PR at temperatures of **(d) **70°C, **(e) **90°C and **(f) **110°C.

However, hot-embossing revealed a drawback in the following holography process due to the possibility of PR degradation during the elevated heating. To minimize the polymer degradation, hot-embossing parameters needed to be initially optimized. Generally, polymers represent three types of thermo-rheological behaviours depending on temperature, e.g., glassy region below glass transition temperature (*T_g_*), visco-elastic region above *T_g _*and viscous region at a temperature sufficiently higher than *T_g _*[20]. Since polymer materials can be thermally decomposed at the viscous region temperature, we carried out hot-embossing - changing from low to relatively high temperatures - to observe the temperature effect on the PR property. Hot-embossing with a PR (AZ nLoF 2020, Clariant, 1.4 μm thickness) was conducted at different temperatures of 70°C, 90°C and 110°C under a pressure of 4.7 bar; the results of each case were shown in Figure 3.

At 70°C, protruded rim shapes at the edges and mounded trench bottom are observed morphologically in the surface profile and seen with different colours in the microscopic image (Figure 3a). Having the master stamp pressed into the PR at the visco-elastic region temperature, the polymer started to flow vertically along the stamp sidewall, eventually resulting in protruded rim shapes. Additionally, an interesting shape of the mounded shape at the trench bottom appeared. As high stresses, which can be released evenly by polymer flow laterally at enough high temperature, are built up and maximized at the center of stamp intrusion at 70°C, the polymer layer in that region is likely to recover to its original shape elastically to dissipate the local stress during the stamp demolding, resulting in the mounded trench bottom [20,21].

As temperature increased beyond the visco-elastic region temperature, elastic response decreased. Meanwhile, plastic response dominated. At temperatures of 90°C and 110°C, polymers became fluidic enough to enhance lateral filling under a pressure of 4.7 bar, resulting in inversely replicated features with vertical sidewalls that were seemingly appropriate for the subsequent phase-shift mask effect, as shown in Figure 3b and 3c.

Above thermally-treated samples at different temperatures were subjected to the single exposure hologram lithography for line patterning; Figure 3d, e and 3f show the cross-sectional views of each sample in the flat region after development. Interestingly, at 110°C, PR material was thermally degraded; the chemically active compound within the PR material was thermally influenced, resulting in unwanted dissolution during the development process at the edges of the exposed regions. In comparison, at 70°C and 90°C, such thermal degradation was not observed. Considering thermal effects on the polymer filling and degradation, hot-embossing was performed at 90°C and 4.7 bar for 10 min afterwards.

In addition, the effects of embossed pattern height on the phase-shift mask phenomenon were investigated. The phase-shift mask of light was represented by *φ *= 2π*Δnh*/*λ*, where *λ *is the light wavelength, 325 nm, Δn is the refractive index difference, 0.63, between air and the phase-shift masking material (PR, refractive index: 1.63), and h is embossed pattern height. Three stamps with 90 nm, 130 nm and 260 nm relief heights - theoretically corresponding to phase-shift mask of π/3, π/2 and π, respectively - were fabricated. Stamps with a feature of 'Y' shape were fabricated on a silicon substrate by the conventional photolithography and subsequent dry-etching.

**Figure 4:**
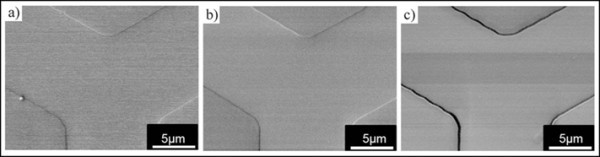
**The effect of relief height on phase-shift phenomenon**. FE-SEM images after UV flood exposure at an energy of 14.5 mJ/cm^2 ^and development with the embossed PR at 90°C by the stamps having a relief height of **(a) **90 nm, **(b) **130 nm and **(c) **260 nm.

The PR patterns embossed with above stamps with different relief heights were then exposed to the diffracted laser light through a 10 μm pin-hole without interference at a total energy of 14.5 mJ/cm^2^. All experimental conditions including the development process were identical except the stamp relief height. SEM images of each case were compared in Figure 4. In case of relief height of 90 nm and 130 nm, the edges of PR pattern were not developed, which means that the lights were not destructively interfered sufficiently and exposed the edges with some intensity because the phase-shift mask is not the integer multiple of π, *n*π. On the contrary, with the relief height of 260 nm that is corresponding to π phase-shift mask, trench line patterns were observed at the very edge of the PR reliefs after development, demonstrating that the embossed feature itself could function as a phase shifter, replacing the conventional PDMS phase-shift mask.

**Figure 5:**
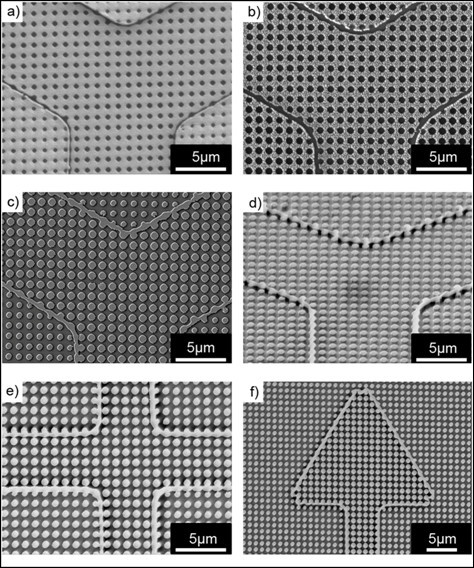
**FE-SEM images of patterns at each step of TAHL process with a stamp having a relief height of 260 nm**. Afterwards **(a) **development, **(b) **oxygen plasma treatment for 600 s, **(c) **Cr lift-off, and **(d) **reactive ion etching, Cr mask removal (tilt view). SEM images of nanoscale silicon line defects along the **(e) **cross and **(f) **arrow edges.

Interference lithography was finally performed with the embossed samples to fabricate periodic hole patterns and line-defects simultaneously by combining phase-shift mask phenomena and periodic exposure. The sample was exposed to the interfered lights with an energy of 7.25 mJ/cm^2 ^twice with 90 degree rotation, equivalent to total energy of 14.5 mJ/cm^2^, at an incident angle of 9.35° for a square lattice of holes. Each exposure time was 10 s. After post-exposure baking and subsequent development, periodic hole patterns with a diameter of 400 nm at a 1 μm pitch were obtained along with line-defects at the PR relief edges with a linewidth of 150 nm (Figure 5a). Oxygen plasma treatment was then performed for 600 s to remove any remaining residual PR at the bottom of patterns. Geometric hole diameter and line-defect width were also affected during the oxygen plasma treatment. Accordingly, hole diameter and line-defect width became 600 nm and 250 nm, respectively, after oxygen plasma treatment as shown in Figure 5b.

After Cr deposition and subsequent lift-off, the remaining Cr pattern was used as an etching-mask while transferring the patterns into a underlying silicon substrate by reactive ion etching (Figure 5c). The tilted SEM image of Figure 5d shows silicon pillars and walls with a height of 300 nm after Cr mask removal. In addition, both cross and arrow patterns were designed and subjected to TAHL process; then, as shown in Figure 5e and 5f, line-defects were faithfully formed along the edges, implying that arbitrary nanoscale line shapes could be easily fabricated by TAHL process once a master stamp with photo-lithographically defined microscale features is prepared.

## Conclusion

This study demonstrated an innovative technique combining holographic lithography and phase-shift mask lithography. Double exposure of PR patterns with an appropriate height to interfered laser lights produced periodic features together with designed functional lines at nanoscale simultaneously. Hot-embossing technique generated the PR patterns with vertical side walls which enabled phase-shift mask effect at the edge of features during the subsequent interference exposure. The shape of nanoscale line-defects can be faithfully incorporated within the periodic structure at the edge of microscale PR patterns. In a long-term perspective, the TAHL technique could be applicable for mass production of photonic devices with functional defects due to its rapid, simple, cost-effective and large area feasible process if further developments regarding alignment of defects to the periodic structure are achieved.

## Methods

### Hot-embossing lithography

Prior to hot-embossing, an anti-sticking self-assembled monolayer (SAM), which reduced the surface energy of silicon master stamp, were employed to enhance stamp demolding from the embossed PR [22]. The silicon substrate was cleaned with RCA-1 (NH_3_/H_2_O_2_/H_2_O) and subsequently treated with acetone, isopropyl alcohol and deionized water for 10 min, then dried with a nitrogen gas. A negative tone PR (AZ nLOF 2020, Clariant) was spin-coated at 3000 rpm for 30 s to have a 1.4 μm thickness on top of an adhesion promoter, HMDS (Hexamethyldisilane, Fluka, Sigma-Aldrich, St. Louis, Mo, USA). Soft baking at 110°C for 1 min was followed in order to remove the residual solvent. The stamp was then placed on the PR-coated substrate and subjected to a pressure of 4.7 bar at a particular temperature for 10 min.

### Holographic lithography

A 325 nm He-Cd laser was used as a light source with an intensity of 0.725 mW/cm^2^. To fabricate the square lattice of holes, double exposure was executed on the embossed PR by rotating 90° before the second exposure. After exposure, post-exposure baking at 115°C for 1 min on a hot plate and the following 2 min development with a MIF 300 developer solution were performed to define patterns. The sample was finally baked at 145°C for 5 min.

### Pattern transfer to the silicon substrate

After development, 20 nm thick chromium (Cr) metal was deposited by an electron beam evaporator. The following 30 min lift-off process with EKC-830 (Dupont Electonic Technologies, Hayward, CA) at 80°C left Cr pattern as an etching mask against the subsequent reactive ion etching to transfer the Cr patterns into the underlying silicon substrate under conditions of 25 sccm of CHF_3_, 2 sccm of O_2_, 20 mTorr and a RF power of 100 W for 200 s. After removal of the Cr mask with an etchant (C-7, Cyantek), silicon features with a height of 300 nm were obtained.

## Competing interests

The authors declare that they have no competing interests.

## Authors' contributions

KSK designed the hologram lithography set-up and BYC initiated this project. BYC carried out the experiments with contribution from YP and GHL. BYC and YP equally contributed to this manuscript. Every work was supervised by GYJ. All authors read and approved the final manuscript.

## References

[B1] Joannopoulos JD Villeneuve PR Fan S Photonic crystals: Putting a new twist on light Nature 1997 386 143 149 10.1038/386143a0

[B2] Yablonovitch E Inhibited spontaneous emission in solid-state physics and electronics Phys Rev Lett 1987 58 2059 2062 10.1103/PhysRevLett.58.2059 10034639

[B3] Yablonovitch E Photonic band-gap structures J Opt Soc Am 1993 10

[B4] Scherer A Painter O Vuckovic J Loncar M Photonic crystals for confining, guiding, and emitting light IEEE T Nanotechnol 2002 1 4 11 10.1109/TNANO.2002.1005421

[B5] Vlasov YA Bo X Sturm JC Norris DJ On-chip natural assembly of silicon photonic bandgap crystals Nature 2001 414 289 293 10.1038/35104529 11713524

[B6] Noda S Tomoda K Yamamoto N Chutinan A Full three-dimensional photonic bandgap crystals at near-infrared wavelengths Science 2000 289 604 606 10.1126/science.289.5479.604 10915619

[B7] Kennedy SR Brett MJ Toader O John S Fabrication of Tetragonal Square Spiral Photonic Crystals Nano Lett 2002 2 59 62 10.1021/nl015635q

[B8] Campbell M Sharp DN Harrison MT Denning RG Turberfield AJ Fabrication of photonic crystals for the visible spectrum by holographic lithography Nature 2000 404 53 56 10.1038/35003523 10716437

[B9] Jeon S Park J Cirelli R Yang S Heitzman CE Braun PV Kenis PJA Rogers JA Fabricating complex three-dimensional nanostructures with high-resolution conformable phase masks Proc Natl Acad Sci 2004 101 12428 12433 10.1073/pnas.0403048101 15314211 PMC515078

[B10] Juntao L Yikun L Xiangsheng X Peiqing Z Bing L Li Y Gershon K Daniel J Kam SW Yongchun Z Fabrication of photonic crystals with functional defects by one-step holographic lithography Opt Express 2008 16 12899 12904 10.1364/OE.16.012899 18711529

[B11] Braun PV Rinne SA Santamaria F Introducing defects in 3D photonic crystals: State of the art Adv Mater 2006 18 2665 2678 10.1002/adma.200600769

[B12] Freymann GV Ledermann A Thiel M Staude I Essiq S Busch K Wegener M Three-dimensional nanostructures for photonics Adv Funct Mater 2010 20 1038 1052 10.1002/adfm.200901838

[B13] Loncar M Doll T Vuckovic J Scherer A Design and fabrication of silicon photonic crystal optical waveguides J Lightwave Technol 2000 18 1402 1411 10.1109/50.887192

[B14] Notomi M Shinya A Yamada K Takahashi JI Takahashi C Yokohama I Structural tuning of guiding modes of line-defect waveguides of silicon-on-insulator photonic crystal slabs IEEE J Quantum Elect 2000 38 736 741

[B15] Cho C Roh YG Park Y Jeon H Lee BS Kim HW Choe YH Photonic crystal slab waveguides fabricated by the combination of holography and photolithography Jpn J Appl Phys 2004 43 1384 1387 10.1143/JJAP.43.1384

[B16] Sun HB Nakamura A Kaneko K Shoji S Kawata S Direct laser writing defects in holographic lithography-created photonic lattices Opt Lett 2005 30 881 883 10.1364/OL.30.000881 15865386

[B17] Scrimgeour J Sharp DN Blanford CF Roche OM Denning RG Turberfield AJ Three-dimensional optical lithography for photonic microstructures Adv Mater 2006 18 1557 1560 10.1002/adma.200502286

[B18] Rogers JA Paul KE Jackman RJ Whitesides GM Using an elastomeric phase mask for sub-100 nm photolithography in the optical near field Appl Phys Lett 70 2658 2660

[B19] Paul KE Breen TL Aizenberg J Whitesides GM Maskless photolithography: Embossed photoresist as its own optical element Appl Phys Lett 1998 73 2893 2895 10.1063/1.122621

[B20] Scheer HC Bogdanski N Wissen M Issues in nanoimprint processes: The imprint pressure Jpn J Appl Phys 2005 44 5609 5616 10.1143/JJAP.44.5609

[B21] Scheer HC Bogdanski N Wissen M Konishi T Hirai Y Polymer time constants during low temperature nanoimprint lithography J Vac Sci Technol B 2005 23 2963 2966 10.1116/1.2121727

[B22] Jung GY Li Z Wu W Chen Y Olynick DL Wang SY Tong WM Williams RS Vapor-phase self-assembled monolayer for improved mold release in nanoimprint lithography Langmuir 2005 21 1158 1161 10.1021/la0476938 15697253

